# What Makes Mobile Banking Difficult for Older Adults? A Multi-Layer Usability Investigation

**DOI:** 10.34172/hpp.44884

**Published:** 2026-06-06

**Authors:** Elahe Amouzadeh, Iman Dianat, Javad Faradmal, Maryam Khalili, Mohammad Babamiri

**Affiliations:** ^1^Department of Ergonomics, Hamadan University of Medical Sciences, Hamadan, Iran; ^2^Department of Occupational Health and Ergonomics, Tabriz University of Medical Sciences, Tabriz, Iran; ^3^Modeling of Noncommunicable Diseases Research Center, Institute of Health Sciences and Technologies, Hamadan University of Medical Sciences, Hamadan, Iran; ^4^Department of Biostatistics, School of Public Health, Hamadan University of Medical Sciences, Hamadan, Iran; ^5^Department of Industrial Design, College of Fine Arts, University of Tehran, Tehran, Iran; ^6^Department of Ergonomics, Research Center for Health Sciences, Institute of Health Sciences and Technologies, Hamadan University of Medical Sciences, Hamadan, Iran

**Keywords:** Application, HCI, Health, Elderly, SMASH, Usability, User experience

## Abstract

**Introduction:**

This study evaluates the usability, accessibility, and ergonomic design of six widely used Iranian mobile banking applications for older adults (aged 60 and above) to enhance digital inclusion and promote healthy aging through improved financial access.

**Methods:**

An *explanatory sequential* mixed-methods approach was utilized, integrating quantitative usability testing with the System Usability Scale (SUS) and qualitative heuristic evaluation using the Smartphone’s Usability Heuristics (SMASH) framework. Five Human-Computer Interaction (HCI) experts evaluated the applications in a controlled lab setting, while 1,200 older adults from diverse backgrounds participated in usability testing and semi-structured interviews. Statistical analysis involved ANOVA with post hoc Least Significant Difference (LSD) tests to compare SUS scores across applications and age groups, complemented by thematic analysis of qualitative data.

**Results:**

Heuristic evaluation identified 135 usability issues, with error prevention (mean severity: 2.27) and visibility of system status (mean severity: 2.22) being the most critical. SUS scores varied significantly (F(5, 1194)=42.36, *P*<0.001), ranging from 27.50 (SD=7.18) for Bank E to 59.02 (SD=7.94) for Bank A. Younger older adults (60–63 years) reported higher SUS scores (M=54.2) than those aged 72–75 (M=45.3, *P*<0.001). Qualitative findings highlighted cultural mismatches and cognitive overload as key barriers.

**Conclusion:**

Poor usability in mobile banking apps widens the digital divide for older adults, hindering financial independence. Tailored designs with simplified interfaces, cultural relevance, and enhanced error prevention can improve digital health literacy, support equitable access and promote mental and social well-being among aging populations.

## Introduction

 Despite the growing shift toward digital financial services, many older adults face significant challenges in accessing and utilizing mobile banking applications effectively. These difficulties are not merely technical; they reflect a deeper misalignment between app design and the cognitive, sensory, and ergonomic needs of elderly users. Addressing these usability gaps is increasingly critical in a world where the global population aged 60 and older is expected to double.^1^ The lack of correlation between age and mobile banking adoption suggests usability barriers affect all ages, particularly older adults.^2^ User-centered design, incorporating end-user preferences such as accessibility and simplified interfaces, is critical for enhancing the usability of digital health platforms for diverse populations, including older adults^3^ Challenges in using digital technologies can significantly impact the social well-being and mental health of older adults. Depicted that anxiety related to ICT use undermines older users’ confidence and performance, limiting their independence and increasing psychological strain. ICT-related anxiety reduces older adults’ confidence, participation, and independence, increasing psychological strain and social withdrawal. Older individuals sometimes require more time to complete activities; therefore, banking apps should adjust their response times to accommodate slower processing rates.^4^ Design modifications, such as simplified displays, enlarged text and button sizes, and adjusted icon shapes, have been proposed to make e-banking apps more effective and user-friendly for older adults, ultimately improving their acceptance and ease of use.^5^ As (Ismatullaev et al. 2022) point out, while human factors are often considered in the design of products and services for the elderly, these considerations are often overlooked.^6^ Traditional and face-to-face relationships are valued in Iranian culture, which can make it difficult for the elderly to adapt to digital connections.^7^ There could be a digital divide in Iran if older people are less accustomed to digital goods and services.^7^ Although Western seniors are typically more acclimated to digital products, several substantial impediments exist. Seniors and other significant segments of the EU population do not utilize the internet efficiently; some even harbor negative attitudes towards it.^7^ Any digital product or service designed for the elderly must consider cultural differences in behavior and emotional needs. Ensuring that digital interfaces are accessible and easy for nontechnical people is essential. Addressing cultural and emotional needs improves digital product design for older adults.^8^ By understanding these cultural differences and addressing the emotional and social needs of the elderly, digital products and services can be better designed to improve their quality of life.^9^ Usability is an important issue that should be considered in product/service evaluation.^10^ HE (Heuristic Evaluation)is a low-cost method that enables evaluators to identify usability issues using a set of criteria. It is practical and can detect approximately 75% of usability problems.^11^

 HE is a cost-effective, adaptable method for evaluating mobile apps, improving interfaces for seniors,^12^ The 12 usability criteria of the SMASH (Smartphone’s usability Heuristics).Fewer studies have focused on this method and its wider dimensions for measurement.The SUS (System Usability Scale) questionnaire is a widely recognized tool for assessing the usability of various applications,^10^ To gain a complete understanding of elderly users’ interaction with banking apps, a mixed-methods approach is essential—combining quantitative metrics with qualitative insights into emotions, behaviors, and cultural context^13^ This is particularly applicable in Iran, where older people can depend on family support, appreciate interpersonal contact, and hold a distrust of technology. By combining both types of data, researchers gain a deeper understanding of barriers and can develop culturally relevant solutions to enhance access and usability. The research assesses six Iranian banking applications based on expert and user insights. Through the implementation of mixed usability methodologies, such as Heuristic Evaluation and the SUS questionnaire, the research aims to establish feasible solutions that increase digital inclusion and promote independent, healthy aging.

## Materials and Methods

###  Study design and setting

 This explanatory sequential mixed-methods design, with the qualitative heuristic evaluation phase providing initial insights to guide quantitative usability testing and integration via merged thematic and statistical analyses to explain usability barriers. The selected six banking applications were chosen based on data from the Central Bank of Iran, which identified them as having the highest user base and national distribution across both public and private sectors. This ensured a representative sample of user experience across diverse platforms, based on their vast user base and the diversity of users’ educational, professional, and digital literacy levels. These applications included those from Mehr-Iran (A), Melli (B), Refah Bank (C), Mellat (D), Tejarat (E), and Saderat (F). The selection included three public (Mehr-Iran, Melli, Refah Bank) and three private (Mellat, Tejarat, Saderat) banks to ensure a comprehensive analysis of the experiences of elderly users.

###  Procedure 

 A *explanatory sequential *mixed-methods design was employed, beginning with a qualitative heuristic evaluation conducted by HCI experts, followed by quantitative user testing using the System Usability Scale (SUS) and qualitative semi-structured interviews with older adults.^13^ Therefore, this study was conducted in two phases: an expert evaluation through an exploratory method and an assessment of real users using the SUS questionnaire. In the first phase, five experts in the field of human-computer interaction (HCI) were selected based on their qualifications and experience to evaluate banking applications using Nielsen’s ^12^ principles. ^14^

a) SMASH is a set of 12 usability principles designed to evaluate mobile interfaces, emphasizing accessibility and user-friendliness for diverse populations, including older adults (Inostroza et al., 2016). The SMASH framework, comprising 12 usability heuristics tailored for smartphone interfaces 15 was used to categorize usability violations identified through Nielsen’s 12 principles. Each violation was mapped to a SMASH heuristic, and experts assigned a severity score (0–4) based on Nielsen’s scale, enabling both qualitative categorization and quantitative prioritization of issues. SMASH scoring: Experts rated violations 0–4 (0 = no issue; 4 = critical) per heuristic (Table 1).
b) GHQ-28 The 28-item (GHQ-28), one of the most validated tools, was used to evaluate the participants’ mental health. The acceptable mental health assessment score threshold was 23.^15^ GHQ-28: Total score 0–84; ≥ 23 indicates good mental health (subscales: somatic, anxiety, social dysfunction, depression. The System Usability Scale (SUS), a 10-item questionnaire rated on a 5-point Likert scale (1 = strongly disagree to 5 = strongly agree), was used to measure usability. The validated Persian version of the SUS has demonstrated good reliability (Cronbach’s alpha = 0.85) and validity.^16,17^ SUS scores range from 0 to 100, with higher scores indicating better perceived usability. The SUS score was computed by summing item contributions as follows: for odd-numbered items (1, 3, 5, 7, 9), the contribution equals the scale position minus 1; for even-numbered items (2, 4, 6, 8, 10), the contribution equals 5 minus the scale position. The total is then multiplied by 2.5 to yield a final score from 0 to 100.^16^ To identify usability issues in six Iranian mobile banking applications, the SMASH framework was applied. The apps were pre-installed on a standardized Android smartphone (Samsung Galaxy A12, Android 11) to ensure consistent display and interaction conditions. Five Human–Computer Interaction (HCI) experts (three university professors and two industry professionals with >5 years of usability evaluation experience) evaluated the apps in a controlled university laboratory, enabling direct observation during both focus-group and individual sessions. A focus group was conducted to discuss ergonomic challenges and SMASH-related variables and to standardize the evaluation procedure. Each app was evaluated using the 12 SMASH heuristic principles, and identified usability violations were rated for severity using Nielsen’s 0–4 scale (0 = no problem; 1 = cosmetic; 2 = minor; 3 = major; 4 = critical). Inter-rater reliability was assessed using Fleiss’ kappa (κ = 0.78), indicating substantial agreement among the experts.

**Table 1:** The SMASH heuristic principles used in the study.

**The SMASH heuristic principle**	**Description**
Visibility of system status	Ensures that the app provides users immediate feedback on their activities, including distinct indications for procedures like verifying transactions and logging in
Match between the system and real-world	Makes the app’s UI more aligned with notions that older users may understand and use well-known terminology and identifiable iconography
User control and freedom	Provides a safety net for inadvertent selections by enabling users to reverse or cancel activities quickly
Consistency and standards	The app's appearance and terminology should be consistent to satisfy older users accustomed to conventional norms.
Error prevention	Guidance tools, including confirmation dialogs for large transactions, are provided to help users avoid errors.
Recognition rather than recall	Reduces memory strain by making commonly used features easily accessible and automatically populating common data
Flexibility and efficiency of use	To improve navigation for senior users, enable customization features, including rapid access to frequently used transactions and reversible font sizes.
Esthetic and minimalist design	Evaluate how well the program completes tasks rapidly, providing seasoned user shortcuts without making it difficult for new users
Help users recognize, diagnose, and recover from errors	Keeps things simple and shows what is necessary; busy screens might confuse older users.
Help and documentation	Provide clear error messages and recovery instructions to help users understand and fix errors.
Customization options	Provide convenient and pertinent assistance elements, such as tutorials and help centers, for frequent activities undertaken by senior users.
Physical ergonomics and interaction	Create an app with big buttons and basic motions that are easy to use while considering user physical constraints

 In the second phase, 1,500 older adults approached via community, 1,200 (aged 60 and above) consented and met inclusion criteria. with at least 1 year of experience using smartphones and banking apps participated. Participants were randomly distributed across apps based on primary use: App A (n = 220, 18.3%), B (n = 210, 17.5%), C (n = 200, 16.7%), D (n = 190, 15.8%), E (n = 180, 15.0%), F (n = 200, 16.7%). For interviews, 10 participants per app (n = 60 total) were purposively selected for depth Each participant used only their primary mobile-banking app and had good mental health (GHQ-28 score ≥ 23). Cluster random sampling ensured geographic and demographic diversity, with 841 men (70%) and 360 women (30%) across Tehran. Participants, recruited from the community and health centers, reflected diversity in gender, education, retirement job, daily mobile use, and computer experience. Additionally, 60 participants (10 per app) were purposively selected from this cohort for semi-structured interviews to explore usability issues in depth; these were conducted at community or health centers, lasting 20–30 minutes each.

 Sample size: Quantitative phase powered via GPower for ANOVA (n = 1,200; α = 0.05, power = 0.90, f = 0.25). A medium effect size (f = 0.25) was assumed based on Cohen’s conventional guidelines for ANOVA and previous usability studies. Qualitative: n = 60 for thematic saturation. Sampling: Cluster-random, with clusters as 6 Tehran districts (randomly selected from 22), stratified by demographics within clusters. The sessions were audio-recorded, transcribed word for word, and analyzed using thematic analysis^18^ Additionally, 60 participants were purposively selected for semi-structured interviews to explore usability challenges in depth. Interviews were conducted at health centers or community centers, each lasting 20–30 minutes. Sessions were audio-recorded, transcribed verbatim, and analyzed using thematic analysis following Braun and Clarke’s (2006) six steps: familiarization, coding, theme searching, reviewing, defining, and reporting.^18^ Two independent coders analyzed the transcripts using MAXQDA 2022 software. Discrepancies were resolved through discussion and consensus. No member checking was conducted due to time constraints

###  Statistical analysis

 There are two primary statistical methods: comparison tests and correlation analysis. To compare means and identify significant differences between groups, analysis of variance (ANOVA) and the Least Significant Difference (LSD) test for post hoc analysis were used.

 Normality was assessed using the Kolmogorov-Smirnov test, and the homogeneity of variances across subgroups was examined using Levene’s test. SUS scores (KS: *P* = 0.12; Levene’s: *P* = 0.08, both non-significant. These tests were applied to examine mean differences in the ^18^ (SUS) scores among various banks and different age groups. Furthermore, Pearson’s correlation analysis was used to investigate the relationship between the duration of mobile phone usage and SUS scores. The researchers drew conclusions about the factors that influence user perceptions of usability by using ANOVA and LSD to compare groups and Pearson correlation to examine relationships. A significance level of alpha = 0.05 was used for all statistical tests.

**Figure 1:**
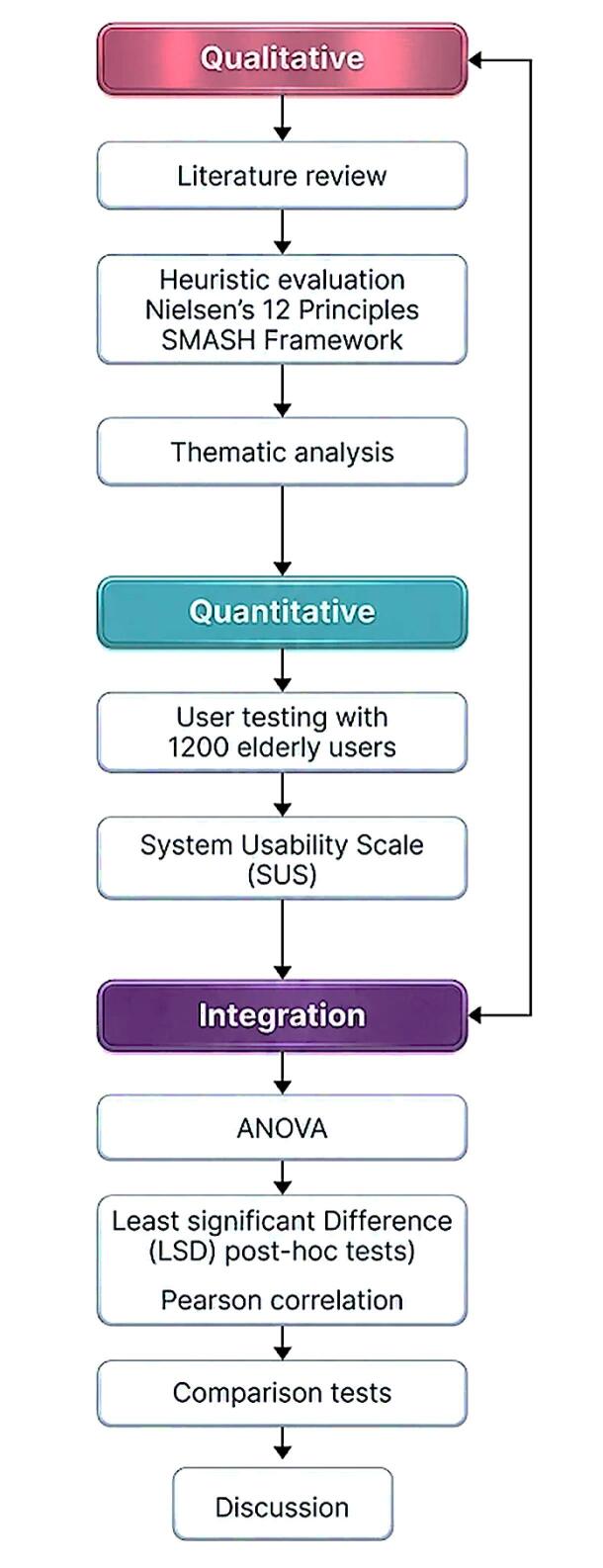
Study Methodology Flowchart for Evaluating Elderly User Experience in Iranian Banking Apps

Figure 1 shows study methodology flowchart for evaluating elderly user experience in Iranian banking apps.

## Results

###  Qualitive

####  Interviews with the elderly participants

**Figure 2:**
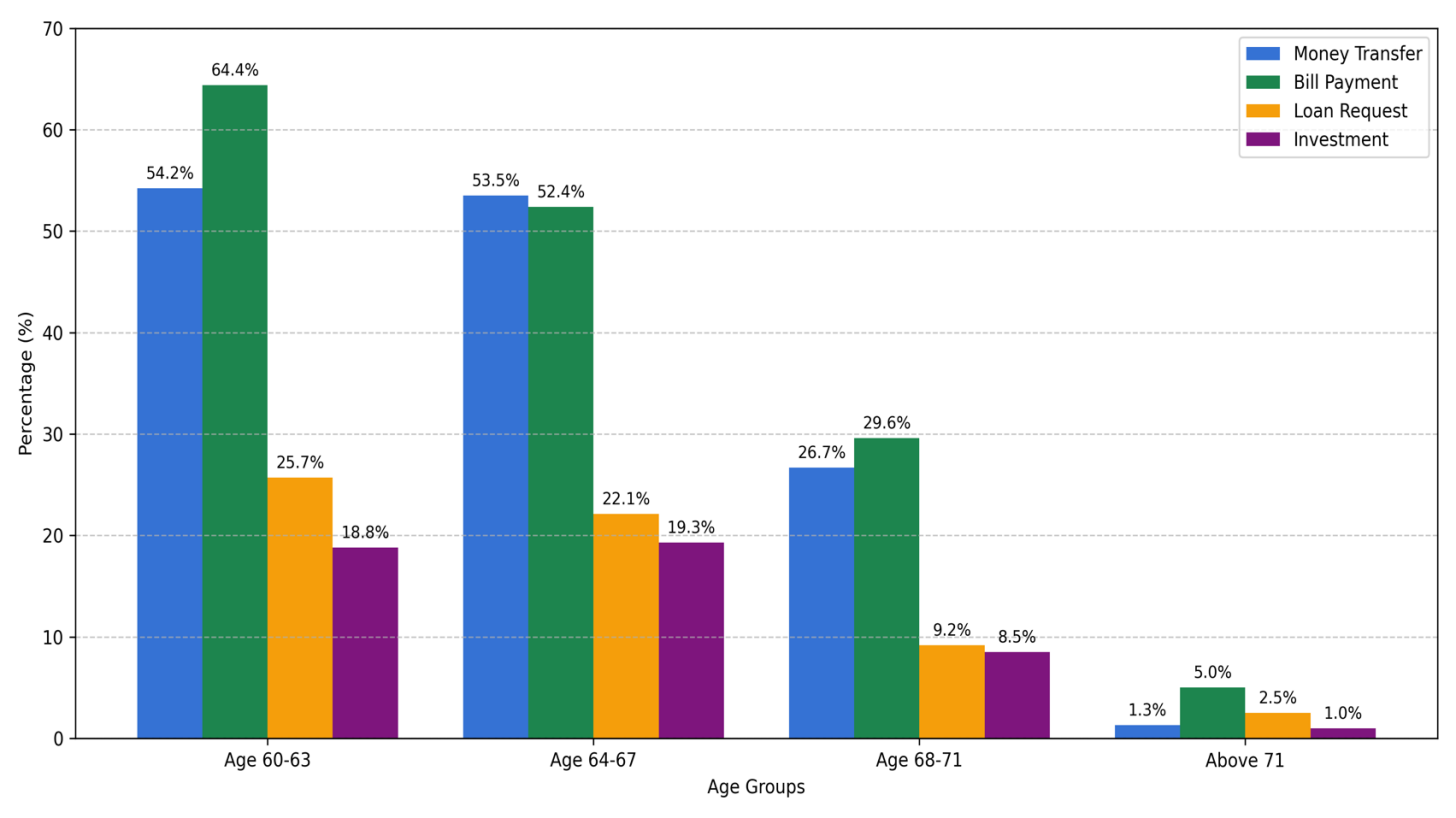
Use of banking services based on the age of participants (n = 1200)

Figure 2 shows the use of banking services based on participant age. Thematic analysis identified three main themes: (1) Navigation Confusion (‘The app changes screens too fast, I don’t know where I am’ – Participant, 68 years), (2) Lack of Feedback (‘I press a button, but nothing tells me if it worked’ – Participant, 72 years), and (3) Unfamiliar Terminology (‘What is PAYA? It’s not explained’ – Participant, 65 years). These themes corroborate expert findings on SMASH principles like ‘Consistency and Standards’ and ‘Match between System and Real World’ (Table 2) The interview results revealed no significant difference between men and women using banking applications (*P* > 0.05). However, there was a highly significant difference within the age groups (*P* < 0.01); thus, younger elderly participants (60-63 years) were more likely to use banking services such as money transfers and bill payments. At the same time, this tendency decreased significantly with increasing age. Money transfer was the most popular banking service among all age groups, with the highest percentage (64.4%) observed among participants aged 60 to 63. Paying bills was also very popular among all age groups, with the highest percentage (53.5%) reported in the 64–67 age group. The loan application was the least used service among all banking services. In general, the use of banking services decreases with age, except for loan requests. Navigation Challenges: Participants reported, “The app’s menus are confusing; I can’t find where to pay bills” (Participant X, aged 65). Feedback Deficiencies: “I don’t know if my transfer went through; there’s no confirmation” (Participant Y, aged 70). Table 2 is summarizing themes and codes (e.g., using MAXQDA outputs) to strengthen the qualitative analysis (Braun & Clarke, 2006).

**Table 2:** Themes and Codes from Thematic Analysis of Semi-Structured Interviews Using MAXQDA (n = 60 Participants; Total Coded Segments = 108)

**Theme**	**Codes/Sub-themes**	**Frequency (out of 108)**	**%**	**Example from Data**
Navigation Confusion	Complex menus, Fast screen changes, Too many steps	38	35.2%	"The app changes screens too fast, I don’t know where I am" (P1, 68 years).
Lack of Feedback	No confirmations, Unclear progress, Loading delays	35	32.4%	"I press a button, but nothing tells me if it worked" (P2, 72 years); "I don’t know if my transfer went through; there’s no confirmation" (P3, 70 years).
Unfamiliar Terminology	Technical jargon (e.g., PAYA/SATNA), Non-local icons, Cultural mismatches	35	32.4%	"What is PAYA? It’s not explained" (P4, 65 years); "The app’s menus are confusing; I can’t find where to pay bills" (P5, 65 years).

###  Nielsen’s principles

####  Severity of problems according to SMASH principles:

 Error prevention (2.27) and consistency and standards (2.12) were ranked as the most severe problems of banking applications, while helping users recognize, diagnose, and recover from errors (1.90), and user control and freedom (1.90) were ranked as the least severe problems.

####  Comparison of different applications

 Regarding user experience, F and E banks’ applications were found to have more problems (particularly ‘error prevention’ and ‘clarity of system status’). A and B banks’ applications had fewer problems (e.g., ‘harmonizing the system with the real world’ and ‘recognizing instead of reminding’) among the evaluated banking applications.

####  Quantitative analysis of problems based on the SMASH principles

 Visibility of system status: With a mean severity score of 2.22, this aspect indicates that elderly users may be confused about recognizing the system’s current state.

 Flexibility and efficiency of use: The mean severity score of 1.98 indicated that banking applications must be improved in providing personalization settings and flexibility to elderly users.

 Esthetic and minimalist design: With a mean severity score of 1.92, elderly users have fewer problems with complex and busy designs, but further design optimization can be helpful.

###  SMASH 

 Supplementary 1 presents the identified problems and proposed solutions for banking applications. A total of 135 problems and their solutions were provided, some of which are presented as follows:

 Summary of High-Severity Usability Problems and Suggested Solutions Grounded in SMASH Principles

**Figure 3:**
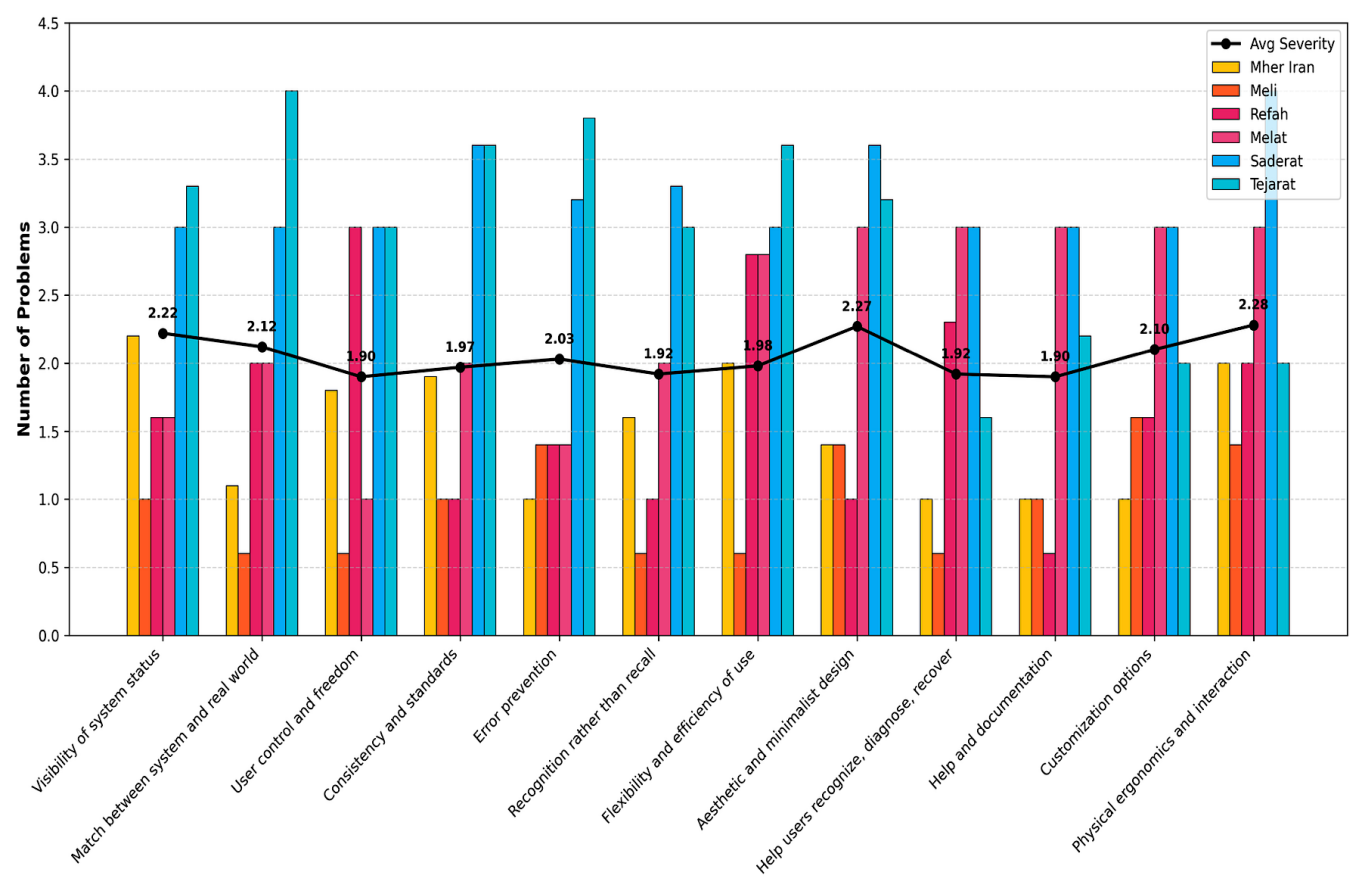
Numbers and severity of detected problems in 6 banking apps

 This section provides a brief overview of high-severity usability problems (severity score ≥ 2, based on Figure 3) detected in the assessment of six Iranian banking apps (A: Mehr-Iran, B: Melli, C: Refah, D: Mellat, E: Tejarat, F: Saderat) by the SMASH framework. Two SMASH principles with critical problems that need to be urgently addressed are presented for each bank, along with their solutions, to maintain consistency with the original material from Supplementary file, Table S1 and Figure 3. Emphasis is placed on problems with the highest severity scores, namely error prevention (2.27) and system status visibility (2.22), to resolve the most critical usability problems for older users.

####  Bank (A)

 Visibility of System Status (Severity: 2.22)

 Problem: Lack of instant feedback regarding transaction status (e.g., “Processing Transaction”) causes anxiety and confusion in older adults.

 Solution: Provide accurate, real-time feedback mechanisms, including on-screen notifications (e.g., “Processing Transaction.”) and graphical indicators like loading animations or progress bars, to increase transparency and minimize user uncertainty.

 Error Prevention (Severity: 2.27)

 Problem: The absence of automated account number verification raises the possibility of incorrect transactions, which lead to user frustration.

 Solution: Incorporate real-time account number validation routines (e.g., digit count and checksum verification) with instant error messages to avert invalid inputs and build user confidence.

####  Bank (B)

 Visibility of System Status (Severity: 2.22)

 Problem: Not displaying the timestamp of the last account update raises suspicions about the validity of the information provided, eroding user trust.

 Solution: Display a visible timestamp (e.g., “Last Updated: May 19, 2025, 14:22”) with financial information to assure users of data freshness and credibility.

 Error Prevention (Severity: 2.27)

 Problem: The lack of warnings for duplicate or invalid transactions leads to financial mistakes and end-user frustration.

 Solution: Create an automated system to identify duplicate or invalid transactions, with immediate warnings displayed to prevent errors before processing.

####  Bank (C)

 Match Between System and Real World (Severity: 2.10)

 Issue: Use of unfamiliar technical terms without explanations confuses elderly users, complicating navigation and interaction.

 Solution: Use simple, commonly understood terms and include tooltips or pop-up definitions for technical jargon to clarify and minimize cognitive load.

 Consistency and Standards (Severity: 2.12)

 Problem: Variability in button designs and color schemes (e.g., green confirm buttons on certain pages and blue on others) disrupts visual consistency and increases the likelihood of errors.

 Solution: Standardize button designs and color schemes throughout the application to ensure visual consistency and reduce user errors.

####  Bank (D)

 Match Between System and Real World (Severity: 2.10)

 Problem: Presenting numeric values without thousand separators (for example, 1000000 instead of 1,000,000) decreases legibility, especially for older users with visual disabilities.

 Solution: Implement uniform numerical formatting with thousand separators (e.g., 1,000,000 Toman) for better readability and understanding.

 Error Prevention (Severity: 2.27)

 Problem: Failure to provide warnings when transactions exceed an account balance result in accidental mistakes and frustration for the user.

 Solution: Utilize real-time balance checking with instant warning messages to prevent transactions with insufficient funds.

####  Bank (E)

 Error Prevention (Severity: 2.27)

 Problem: Lack of automated card number verification raises the possibility of transactional mistakes, especially for older users with poor dexterity.

 Solution: Implement card number validation algorithms (e.g., Luhn algorithm) with real-time error messages to provide precise inputs and minimize transaction failures.

 Visibility of System Status (Severity: 2.22)

 Problem: The absence of any visual or text feedback following transaction confirmation leaves users confused and uncertain.

 Solution: Display distinctive feedback messages (“e.g., “Transaction Successful”) and loading animations to validate transaction status and increase user confidence.

####  Bank (F)

 Consistency and Standards (Severity: 2.12)

 Issue: Inconsistent terminology for the same function creates confusion and hinders user navigation.

 Solution: Standardize terms throughout the application for consistency and to enhance user understanding.

 Error Prevention (Severity: 2.27)

 Problem: Lack of a final confirmatory step for transactions heightens the possibility of accidental transactions, leading to user frustration.

 Solution: Implement a compulsory final confirmation step that displays vital transaction information (e.g., recipient and amount) before processing to prevent mistakes.

 Quantitative Results (Figure 3): The most critical problems detected are error prevention (average Severity: 2.27), visibility of system status (2.22), consistency and standards (2.12), and match between the system and the real world (2.10). These problems are most evident in banks E and F, where urgent design revisions are needed to make the systems more usable for older people. Figure 3 presents the number and severity of detected problems in the six banking applications

###  Quantitative

####  Usability

**Figure 4:**
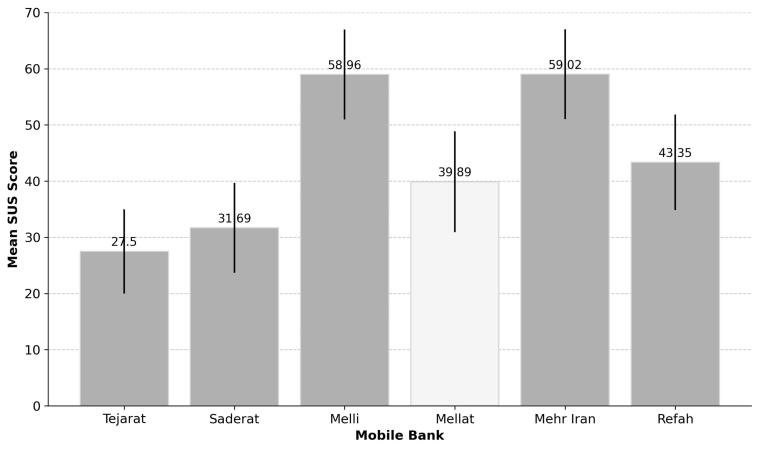
SUS mean Score in 6 banking apps

Figure 4 shows the mean SUS scores of various mobile banks. The banking applications of A (M = 59.02, SD = 7.94) and B (M = 58.97, SD = 7.92) banks had the highest scores, whereas the E (M = 27.50, SD = 7.18) bankapplication had the lowest score.

**Figure 5:**
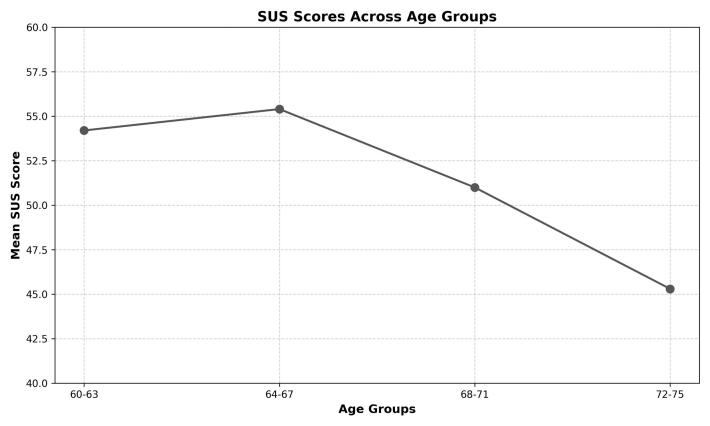
Average SUS score by age group

**Figure 6:**
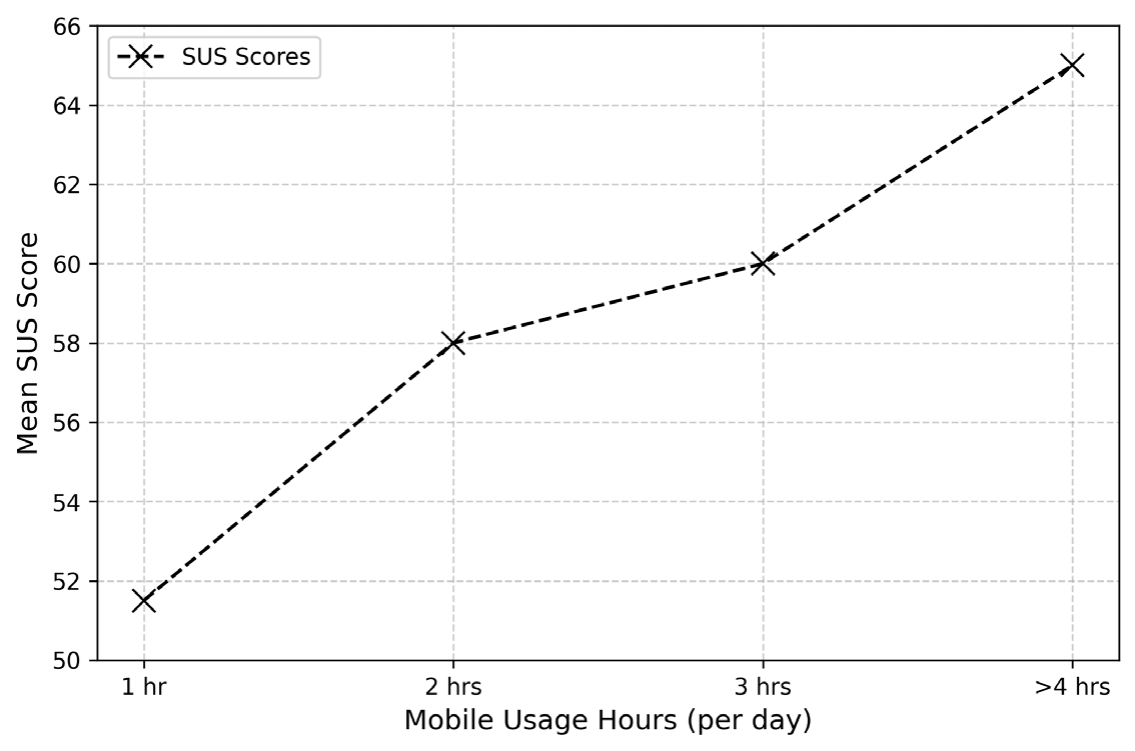
Average SUS score by mobile hours usage

Figure 5 shows the changing trend of the SUS scores according to age group. The 60-63 age group had the highest mean SUS score (54.2), and the scores gradually decreased with increasing age, so the 75-72 age group had the lowest mean SUS score (45.3). Figure 6 Correlation between mobile Correlation between mobile use duration and SUS scores. The results indicated that as the duration of mobile use increased, the mean SUS score also improved. Those using their mobile > 4 hours per day recorded the highest SUS score (65.0), while users with 1 hour of mobile use per day had a lower score (51.5).

Supplementary file, Table S1 shows After adjusting for age, significant differences were found among banking applications in terms of usability. ANOVA revealed significant differences in SUS scores across banks (F(5, 1194) = 127.63, *P* < 0.001), with LSD post hoc tests confirming pairwise differences. For Bank E vs. Bank A, the mean difference in SUS scores was -31.52 (95% CI: -33.10, -29.94, *P* < 0.001), with a large effect size (Cohen’s d = 1.82), indicating substantial usability disparities. To identify these differences, LSD post hoc tests were conducted. E Bank: Performance was significantly worse than all other banks (F, B, D, A, and C). B Bank and A: Did not exhibit significant differences in user satisfaction (*P* = 0.946). D Bank: Outperformed E, A, and C banks in terms of user satisfaction. Bank C: Received significantly higher user ratings than Bank A.

 ANCOVA was conducted with age, education level, and socioeconomic status as covariates. None of the covariates showed significant effects (all *P* > 0.05); therefore, adjusted means were equal to the raw means, and the original pairwise comparison results (Table 3).

**Table 3:** Comparison of Mean Differences among Mobile Banking Apps for SUS total Adjusted for Age

**Groups**	**Mean Difference**	**Confidence Interval (Lower)**	**Confidence Interval (Upper)**	***P*** **-value**
E-F	-4.19	-5.77	-2.61	< 0.001
E-B	-31.46	-33.03	-29.89	< 0.001
E-D	-12.39	-13.98	-10.81	< 0.001
E-A	-31.52	-33.10	-29.94	< 0.001
E-C	-15.85	-17.43	-14.27	< 0.001
F-B	-27.27	-28.82	-25.72	< 0.001
F-D	-8.20	-9.76	-6.64	< 0.001
F-A	-27.32	-28.88	-25.77	< 0.001
F-C	-11.66	-13.22	-10.10	< 0.001
B-D	19.06	17.51	20.62	< 0.001
B-A	-0.05	-1.60	1.49	0.946
B-C	15.61	14.05	17.16	< 0.001
D-A	-19.12	-20.68	-17.56	< 0.001
D-C	-3.45	-5.02	-1.89	< 0.001
A-C	15.66	14.10	17.22	< 0.001

## Discussion

 As demonstrated in this study, an evaluation of banking applications using Nielsen principles revealed several design issues that should be addressed to enhance the user experience for the elderly population. In this regard, the studied banking applications have various design deficiencies, indicating that each has its design considerations. The application of Nielsen’s principles in evaluating product user-friendliness has been confirmed in several studies.^19^ However, the SMASH method offers more advantages with a more detailed and multidimensional evaluation.^20^ Salman HM (2018) used this method to evaluate mobile application use among elderly individuals and identified 27 problems, including cognitive load and guidance.^21^ By more closely examining specific design aspects that affect the experience of the elderly, this method adequately reveals the main challenges of designing these applications for the elderly.

###  Summary of Key Findings

 The SMASH evaluation revealed critical usability issues across the six banking applications, with the highest severity scores for error prevention (mean: 2.27), visibility of system status (2.22), consistency and standards (2.12), and match between system and real-world (2.10) scenarios, as reported in Figure 3. Bank (E) exhibited the lowest usability (SUS score: 40.2), attributed to inadequate transaction feedback and lack of automated validation, while Mehr Iran (A) and Melli (B) scored higher (SUS: 58.2 and 56.9, respectively), reflecting better alignment with elderly users’ cognitive and physical needs. Qualitative findings highlighted complex terminology, inconsistent button designs, poor contrast, and insufficient error recovery mechanisms as key barriers for elderly users. These issues align with the original discussion’s emphasis on cognitive load, color misuse, and ergonomic deficiencies, particularly in E and F Banks.

###  Interpretation of Findings in Context of Literature

 In an evaluation of banking applications, several issues related to the inappropriate use of color were identified, which have a significant impact on user experience. The findings extend prior usability research by applying the SMASH framework, which offers a more granular evaluation than Nielsen’s heuristics^20^ Unlike Salman et al. (2018), who identified 27 usability issues in mobile applications for elderly users, this study highlights design flaws such as the absence of transaction feedback and complex banking jargon, which increase cognitive load and hinder usability These issues resonate with eHealth literacy research, where elderly users’ limited technological proficiency exacerbates navigation challenges.^22^ For instance, the use of unfamiliar terms in C and D Banks mirrors findings by Czaja et al. (2019), who note that complex interfaces widen the digital divide for older adults.^21^

 The Technology Acceptance Model (TAM) offers a theoretical framework for interpreting these results. TAM posits that perceived ease of use and usefulness drive technology adoption.^23^ B Bank’s low SUS score reflects the poor perceived ease of use due to its lack of error prevention and feedback, which deters elderly users. Conversely, A and B Banks’ higher scores suggest better alignment with TAM’s ease-of-use construct, achieved through clearer instructions and reduced cognitive demands.^23^ The inconsistent use of colors and symbols, as noted in the original discussion, aligns with Liu and Wang (2023), who advocate for high-contrast, warm-color interfaces to enhance readability for elderly users with visual impairments. Similarly,^24^ Wong et al. (2018) emphasize the use of culturally relevant symbols to bridge the gap between system design and elderly users’ real-world experiences, supporting the need for simplified, familiar interfaces. ^25^

 Recent studies confirm that older adults’ engagement with digital financial services is directly tied to perceived emotional support, usability simplicity, and trust in digital systems (Wang & Huang, 2024; Alshamrani et al., 2025). These findings align with our study, especially regarding inconsistent feedback and terminology confusion as barriers to digital self-efficacy. A 2025 study by Gómez-López et al. highlights how minimalistic design and culturally localized UI elements significantly enhance older users’ perceived control and reduce anxiety during online transactions.^26^ Moreover, a meta-analysis by Ahmad et al. (2024) emphasized the dual role of mobile banking usability in both financial independence and emotional wellbeing among older adults, particularly in developing countries. Our findings regarding error prevention and lack of confirmation dialogs reflect these challenges. These results suggest that improvements in system responsiveness and error mitigation are essential not only for enhancing user satisfaction but also for promoting digital inclusion, strengthening user trust, and supporting equitable access to digital financial services among aging populations.

 From a health promotion perspective, the identified usability barriers hinder elderly users’ access to financial services, undermining financial inclusion and autonomy. ^23^ The absence of clear error recovery mechanisms, as observed in E and F Banks, aligns with digital equity studies that emphasize the importance of inclusive design in supporting vulnerable populations.^27^ These findings extend the original discussion by linking usability issues to broader societal implications, such as equitable access to digital financial services.

###  Influence of Age and Digital Experience

 The study found a negative correlation between age and usability satisfaction, with users aged 60–63 reporting higher SUS scores (mean: 54.2) than those aged 72–75 (mean: 45.3). This aligns with cognitive load theory, which suggests that age-related declines in working memory increase the difficulty of navigating complex interfaces. ^28^ For example, Tejarat Bank’s technical terms without tooltips exacerbated cognitive strain for older users. Users with over four hours of daily mobile use reported higher SUS scores (mean: 65) compared to those with less than one hour (mean: 51.5), supporting 30 who found that digital familiarity reduces the psychological digital divide in elderly users by enhancing confidence in technology use.^29^

###  Implications for Design of Elder-Friendly Apps

 The findings have significant implications for designing elder-friendly banking applications. First, aligning interfaces with real-world conventions, as suggested by Wong et al. (2018), can reduce cognitive load and enhance eHealth literacy^30^ For example, replacing technical terms with plain language and providing tooltips, as recommended for C and D Banks, can improve comprehension. Second, robust error prevention mechanisms, such as real-time validation, align with digital equity principles by ensuring accessibility for users with limited technological proficiency.^31^ Third, consistent design elements (e.g., standardized button colors) and immediate feedback (e.g., transaction status indicators) enhance perceived ease of use, as per TAM, fostering greater adoption among elderly users.^23^

 In 2024, Kwon and Jeong proposed an adaptive interface framework for aging populations, integrating real-time cognitive load monitoring to dynamically adjust complexity and visual density. Incorporating similar adaptive mechanisms in Iranian banking apps could significantly improve engagement rates among older adults with lower digital literacy. Likewise, Liu et al. (2025) demonstrated that voice-activated guidance systems with culturally familiar terminology increased task completion rates by 34% in users aged over 65, suggesting that future Iranian banking apps should explore such voice-enabled interfaces.

 These implications extend the original discussion by linking usability improvements to broader goals of financial inclusion and digital equity.

## Strengths and Limitations

 The study’s strengths include its use of the SMASH framework, which provided a multidimensional evaluation beyond Nielsen’s heuristics, and its focus on elderly users, a demographic often overlooked in HCI research 21. The qualitative thematic analysis revealed nuanced issues, such as the impact of cultural metaphors on usability, which were underemphasized in the original discussion. However, limitations include the lack of statistical analysis beyond mean severity scores, which could clarify why E Bank underperformed (e.g., through variance or regression analysis). Additionally, the study did not explore cultural variations within Iran, which may influence user preferences, as noted by 33 The reliance on SUS scores without qualitative triangulation of user perceptions limits the depth of insight into user satisfaction.

 limitation worth noting is the lack of examination of emotional and psychological impacts of usability issues. Recent work by Varela & Chen (2025) stresses the importance of evaluating affective responses—such as frustration or anxiety—through biometric or behavioral feedback, especially in elderly digital users. Including such multimodal evaluations in future studies would offer a deeper understanding of user experience and help tailor more inclusive technologies

## Practical Recommendations

 To address the identified usability issues, the following evidence-based recommendations are proposed:

1. Enhance Visibility of System Status: Implement immediate feedback mechanisms, such as loading animations and timestamps (e.g., “Last Updated: July 21, 2025, 01:19”), to reduce user anxiety, particularly for E and F Banks.^24^
2. Strengthen Error Prevention: Integrate real-time validation algorithms (e.g., Luhn algorithm for card numbers) and confirmation prompts to minimize errors, addressing the high severity score (2.27) across all banks.^21^
3. Simplify Language and Design: Use plain language, tooltips for technical terms, and standardized button designs to enhance consistency and real-world alignment, especially for C and D Banks.^25^
4. Reduce Cognitive Load: Provide step-by-step instructions and dropdown menus for repetitive tasks to support users with declining memory, as exemplified by A Bank.^32,33^
5. Improve Physical Ergonomics: Increase button sizes, enhance contrast, and add haptic feedback to accommodate the physical limitations of elderly users, particularly in F Bank^34^ These recommendations align with health promotion and digital equity goals, ensuring that banking applications are accessible and intuitive for elderly users, thereby fostering financial inclusion.

Additionally, the integration of AI-driven customization, as explored by Zhao et al. (2025), allows applications to automatically adjust font size, contrast levels, and input difficulty based on user behavior and age group. These features should be considered in future redesigns of Iranian banking apps to enhance accessibility and foster independent financial management among older adults.

## Competing Interests

 The authors declare that they have no competing interests.

## Ethical Approval

 This study was approved by the Ethics Committee of Hamadan University of Medical Sciences (IR.UMSHA.REC.1401.925).

## Supplementary File

Supplementary File contains Table S1.
