# Supplementary material for: What Makes Mobile Banking Difficult for Older Adults? A Multi-Layer Usability Investigation
Source: Health Promot Perspect. 2026 Jun 6;16(1):74–84. doi: 10.34172/hpp.44884 (PMC13402424; doi:10.34172/hpp.44884)
Supplement: Supplementary file 1 — Supplementary File contains Table S1. [file hpp-16-74-s001.pdf]

Amouzadeh et al, Health Promotion Perspectives 2026;16(1):74-S1
doi:10.34172/hpp.44884
https://hpp.tbzmed.ac.ir

**Table S1. Identified Problems and Proposed Solutions for Banking Applications**

| **Ite** | **Problem description** | **solutions** |
|---|---|---|
| Visibility of system status | P1: Transaction messages are not triggered after clicking on the confirmation button. In most cases, after the completion of a money transfer, the user receives no information indicating that the transaction is being processed. P2: The completion of the transaction is not followed by a visual indication or a message. It does happen that during the execution, no confirming message of the transaction is shown to the user. P3: There is no indication or animation of loading while loading up an account. Nothing displays when account information is loading; there is no bar or loading message, and the user has no idea whether the program is functioning. P4: Connect the number of internet connection status notifications that the user can see. If the internet is disconnected for whatever reason, communications will not be sent, informing the user that there is no connection. P5: The papers have not yet been registered, so the user cannot get information about their status. It is unclear to the user where the request he has registered (such as the application for a loan) is in terms of status (active, looking for assurance, rejected, etc.) status. P6: Users are not correctly shown system failures: When an error occurs, either no error message will come up for the user or the message is displayed that is too general and does not provide detailed information regarding the cause of the error. P7: The update source shall not highlight the time of the last update of the account, which holds up dates. It would leave a question mark on the end user whether their balance is the latest.<br>P8: Alternatively, an unsuccessful message will not be there; however, if that is the case or if the login worked, the users will be in the dark and will remain unaware whether they are logged in.P9: Still, for some kinds of transactions where a set time frame is required for a transaction to be completed, the timer showing how much time is left to finish up the transaction is not included in the system. P10: Even if such a system mode is enabled to improve security, the user will not be notified about it through a message. P11: Similarly, if the focus performer reverses specific configurations, for example, getting a different e-mail than the one currently in use, no such message is given that such a change was made.P12: Not as such goes. No visible aspects appear to be provided to the audience when the picture | S1: Upon clicking the button to confirm a transaction, post a pop-up to confirm the transactio<br>S2: Place a picture or text to mark the transaction as successful.<br>S3: Include any loading animations or a bar illustrating the account loading processes.<br>S4: Provide communication if there is a disconnect to the internet while watching the stream.<br>S5: Send notifications of the requests made: active, pending, and declined requests.<br>S6: Detailed and well-articulated error messages with straightforward information.<br>S7: Ensure the date and time when the account was last updated is easily visible.<br>S8: Use either a success or failure message to log in and update the user.<br>S9: Use a timer to show how much time is left before completing the transactions.<br>S10: Send alerts to users whenever they enable or change further security measures.<br>S11: Send alerts when blue-sensitive email settings are changed.<br>S12: Update progress when a progress bar or visual notification is displayed.<br>S13: Issue notification messages concerning queues and expected times in the banking setting |

| | | |
|---|---|---|
| | progresses, and the application is updated. P13: Lack of any notification messages regarding expected delays in the system: In the situation of a problem or a delay in the central bank system, the user is not informed that transactions may be slow. | |
| Match between the system and real world | P1: Using terms rarely used in conversations, such as PAYA or SATA, without defining them complicates the overall process for regular users P2: Referring to them in strange terms or providing them with words that the everyday person will not understand like making words such as a current account or savings account to general terms. P3: Giving a date format less commonly, like YYYY-MM-DD, which the user may not understand, resulting in them entering the wrong information. P4: Representing approving or rejecting request status with unconventional colors or symbols that are not common. P5: Defining a transaction type or an option for an account in a manner that the user does not recognize from their regular interaction with a banking system or terms in a conventional bank setting. P6: Omitting critical symbols such as Toman or Dollar, which are the local currency symbol in the display which may lead to confusion. P7: Presenting the accounting information with specific limitations or constraints, for example, presenting account balance in thousands or millions where the average person understanding may be limited. P8: Provide no straightforward procedures that are easily understood by the ordinary Tina when one is applying for a loan or trying to make complex deposit scenarios. P9: Avoid not using a language or message that a layperson would understand and consider as being friendly and natural. For example, P10: Forcing clients to use alphanumeric number codes that are difficult to remember instead of sending them an SMS message to their phones as an additional security measure. P11: Originating information irrelevant to most users concerning daily transactions in inappropriate technical terminology. P12: Employ strange graphics instead of simple 'pay,' 'Make a transfer,' and similar commands. P13: There is covariance within money transfers, for example, 'money transfer—national' and 'money transfer—international,' but they do not explain it, leaving users bewildered. P14: Make all the financial values available in numerical figures without the comma separation of the figures for hundreds, thus obscuring large volumes of the figures. | S1: Use the familiar interface to integrate clear text descriptions of terms such as PAYA o<br>instead of using these terms or explaining where they are needed in a tooltip or pop-up.<br>S2: The terms' Checking Account' and 'Savings Account' can be used instead of more technica<br>which makes it easier for people to understand.<br>S3: Allow users to present dates simultaneously in any of the two variations with format exten<br>by default in the dymddam or ddmmdy systems.<br>S4: Any other color may also be used only with specific text speak regarding the use of suc<br>e.g., orange for endorsement may also be marked with the word 'endorsement.'<br>S5: Use simple terms or options for transaction types and include other terms with helpful ex<br>S6: In all cases, optical amounts should indicate local currencies, for example, Toman or Rials<br>regions.<br>S7: In accounting information, mainly when dollars are involved, figures should be provided a<br>number and in commas of thousands.<br>S8: Provide pictures and instructions to avoid lengthy process steps like loan applications.<br>S9: Use pleasant and compassionate messages for the prompts, etc., to make it easy for the<br>understand everything.<br>S10: Alphanumeric codes can be written off and replaced with more straightforward methods<br>sending an SMS message or using biometric features like thumb or face.<br>S11: Sound judgment should be exercised in all such instances to avoid unnecessarily technica<br>S12: In such notices, logos, and images are replaced by more straightforward instructions li<br>or "Transfer" that capture the function the information will likely deliver.<br>S13: Such important features should include "Money Transfer within the Country" and<br>Transfer abroad." |

| | | |
|---|---|---|
| User control and freedom | • P1: Effectively a transaction lockout after a user selects a preference: The user has no option of unselecting a transaction that he selected in error when it is their turn to be processed.<br>• P2: No common back and exit buttons: Users have no straightforward means of moving or clicking backward to the last interface or returning to the main screen on all pages that they went in by error.<br>• P3: Prohibitions on editing transaction data prior to confirmation: A user is not allowed to change details like the sum, the recipient of the transaction, or similar parameters; instead, he/she has to repeat the transaction from the beginning again.<br>• P4: Absence of a final confirmation step when a user wants to make a transaction: The user may initiate a transaction and, through careless actions, be allowed to complete the transaction without confirming.<br>• P5: Lack of ability to go back to the settings before they were changed: The user cannot revert to an old one once they have made changes without trying.<br>• P6: Sending any requests, such as loan or card requests, that cannot be undone: After sending a request, the user cannot rescind or modify it.<br>• P7: Concerns about exit time during emergencies: For example, the need to log out or into an account at a specific time; in most cases, there are no options.<br>• P8: Lack of features to control the information presentation structure: A user is restricted on how he/she wants to present different data, including the number of views and the content of the views.<br>•P9: There is no way to filter the notifications, so the user has no control over which to allow and which to turn off. | S1: Add Undo Transaction Selection; provide a button to undo incorrect transaction selectio [illegible] final processing. This feature will significantly reduce user errors and enhance the over [illegible] experience.<br>S2: Design of general back and exit buttons to improve navigation. Ensure that the buttons [illegible] the previous page and exit to the home page on all pages.<br>S3: Ability to edit information before final transaction confirmation<br>It empowers users with the flexibility to edit transaction details, such as amount and recipient, [illegible] final confirmation, thereby improving their sense of control.<br>S4: Add the final transaction confirmation stage to prevent unwanted errors and display a mes [illegible] the final transaction confirmation before processing is complete.<br>S5: Create a return to previous settings option and be able to restore previous settings after [illegible] with a "return to previous settings" button.<br>S6: Provide the option to cancel submitted requests. You can cancel or edit requests subm [illegible] mistake, such as loans or card issuance.<br>S7: Improve speed and accessibility in emergencies. Design quick exit and entry options for s [illegible] situations.<br>S8: Ability to customize how information is displayed; provide tools to adjust how data are di [illegible] such as the number of views or visible content.<br>S9: Create filters for notifications and add the ability to manage notifications and turn [illegible] messages on or off. |

| | | |
|---|---|---|
| Consistency and standards | P1: Using different terms for the same function: For example, a button in different parts of the application is sometimes displayed as "SORATHESAB" and sometimes as "HESAB," which confuses the user.<br>P2: Different design and appearance of buttons and icons on different pages: The buttons and icons on different pages of the application differ in terms of design and color, which disrupts visual harmony.<br>P3: Different date and time display formats: The date and time in different application parts are displayed in different formats, such as DD/MM/YYYY and YYYY-MM-DD, which can be confusing.<br>P4: Inconsistency in writing style and language: Some pages use formal and informal language, creating inconsistent user experiences.<br>P5: Inconsistent display of account information: Account information, such as balances and transactions, is displayed on some pages with whole numbers and others with rounded numbers.<br>P6: Use different colors for confirm and cancel buttons: On some pages, the confirm button is green, and on others, it is blue, causing inconsistency and potential for errors.<br>P7: Different ways to display errors: In different sections, errors are sometimes displayed using text messages and warning icons.<br>P8: Inconsistency in access icons to the main sections: For example, the "Home" icon is located at the top of the screen on some pages and at the bottom on others.<br>P9: Difference in how the help is presented: On some pages, the help is displayed as a pop-up, and on others, it is presented as a fixed text.<br>P10: Inconsistent use of currency symbols: In some sections, a currency symbol such as "Toman" is used, and in others, only the Toman sign or currency code (IRR) is used.<br>P11: Different login and authentication methods: Some pages require a second password, and others require only the primary password. This confuses users.<br>P12: Use of different names for the same type of account: In some areas, "current account," and in others, "deposit account" refers to the same type of account.<br>P13: Inconsistency in the appearance and function of the navigation bar: In some pages, the navigation bar is fixed; in others, it is moving, or its appearance is different.<br>P14: Inconsistency in the display of financial numbers: On some pages, the numbers are displayed with thousand separators, and on others, the numbers are displayed without separators, making it difficult to read large numbers. | S1: Use consistent terminology across the app to avoid user confusion.<br>S2: Standardize button and icon designs to maintain visual harmony.<br>S3: Ensure consistent date and time formats throughout the app to reduce confusion.<br>S4: Use AI to adjust language styles based on user preferences and gamify the process by re[illegible] consistency in language choices.<br>S5: Display consistent account information in whole numbers or rounded figures fo[illegible] understanding.<br>S6: Use a consistent color scheme to confirm and cancel buttons.<br>S7: Display errors uniformly using either text or icons to improve communication.<br>S8: Use AI to track user navigation patterns and determine optimal placement of acces[illegible] rewarding consistency with points or badges.<br>S9: Use a fixed method to display help, such as pop-ups or fixed text, to improve user experi[illegible]<br>S10: Use a unified symbol for currency to avoid confusion and enhance clarity.<br>S11: Simplify login and authentication methods using AI that adapts based on user beha[illegible] gamifies the process with rewards to realize seamless access.<br>S12: Use consistent account names to eliminate ambiguity and improve user confidence.<br>S13: Standardize the navigation bar layout to enhance usability and make it easier for users to n[illegible]<br>S14: Ensure consistent display of financial numbers, such as using or omitting thousand separ[illegible] improve readability. |

| | | |
|---|---|---|
| Error prevention | • P1: No warning about invalid values: If the user enters an illegal value (such as negative numbers or letters) in the amount field, no error message or warning will be given.<br>• P2: No validation of account numbers: When entering the destination account number, the program will not immediately detect the error if the number is incorrect or invalid.<br>• P3: Failure to match the input card number: If the user enters the card number incorrectly, the program cannot match or check its correctness; thus, the transaction may be performed incorrectly.<br>• P4: No warning message if the transaction is not confirmed: If the user presses the submit button by mistake without final confirmation, no warning will be given to prevent this error.<br>• P5: Not displaying transaction limits before action: If transactions with an amount exceeding the daily or monthly limit are made, these limits will not be displayed before action.<br>• P6: The possibility of making repeated transactions by mistake: If the user makes a transaction again by mistake, the program will not inform them that the transaction is repeated.<br>• P7: No warning for insufficient balance: If the user tries to send more than their balance, the app will not immediately notify them.<br>• P8: No warning for critical times: If transactions are performed during the banking system's closing times or at the end of the day, the application does not warn the user that the transaction may be delayed.<br>• P9: Failure to check the security code before confirming the transaction: If the user enters the security code incorrectly, the application will not recognize the error at that moment and will allow the error to occur.<br>• P10: No confirmation message for sensitive settings: When changing sensitive settings, such as passwords, the program does not ask for reconfirmation from the user, and the user may change the settings by mistake.<br>• P11: The possibility of not saving the destination card number after the transaction: The program does not allow the user to easily save the number of cards to which money has just been sent, and the user must re-enter the number.<br>• P12: There is no automatic detection system for duplicate information. If the user accidentally enters duplicate information (such as re-registering a bank card), the system will not display a warning.<br>• P13: The possibility of making a mistake in choosing the account type for transferring funds: If the user chooses the wrong account type, he/she will not receive any warning to match the source and destination accounts.<br>• P14: No warning for using old information in transactions: If the user uses old information (such as account number or payment date), the application will not notify him or her of any change in the information. | S1: Validate inputs in numeric fields. Solution: Real-time data validation is implemented f[illegible] such as transaction amount, which invalidate invalid (i.e., letters or negative numbers) va[illegible] sending an error message.<br>S2: Automatic validation of account numbers solution: We implement algorithms to check the [illegible] of account numbers (such as checking the number of digits and the checksum) and display [illegible] message immediately after entering an invalid number.<br>S3: Card number matching algorithm solution: This algorithm implements card number va[illegible] algorithms (e.g., the Luan algorithm) and displays an error message if the card number is [illegible] incorrectly.<br>S4: Final confirmation message for transactions Solution: Add a final confirmation step wit[illegible] message for the user that includes crucial transaction information, such as the amount, destinat[illegible] number, and account holder's name.<br>S5: Display transaction limits prior to taking action. Solution: Users should be informed ab[illegible] and monthly transaction limits before performing financial functions.<br>S6: Prevent duplicate transactions. Solution: Design a system to identify duplicate transact[illegible] display a warning to the user that a similar transaction has already been made.<br>S7: Real-time warning for insufficient balance Solution: After inputting the balance, verify t[illegible] account balance and show a message if the balance is insufficient.<br>- S8: Warning for sensitive times: Note and advise on sensitive periods (like day end or ban[illegible] availability) that could lead to processing transaction delays.<br>- S9: Check the security code before confirming the transaction. Solution: Use real-time ver[illegible] of security codes and prevent the process from continuing if entered incorrectly.<br>S10: Request re-verification for changing sensitive settings Solution: Include a re-authentica[illegible] (e.g., username and password reconfirmation, SMS code verification) that must be perform[illegible] changing a sensitive setting (e.g., changing the password).<br>- S11: The destination card number is saved after the transaction. Solution: Implement a fe[illegible] store the recipient card number at the end of each purchase, allowing the user to delete or mo[illegible] number.<br>- S12: Automatic duplicate information detection system: This system identifies the pr[illegible] redundant data (like re-entering credit card details) and alerts the user.<br>S13: Alert for matching account types in money transfers. Solution: We verify whether the so[illegible] the account's destination types are correct and display a warning message if they differ.<br>S14: Warn about outdated information. Solution: Researchers have developed a system that [illegible] the validity of information (e.g., account number and relevant dates) and alerts the user if it ha[illegible] to be valid.<br>S15: Preview transaction information prior to submission. Solution: Display a preview page [illegible] the complete transaction details (payer, recipient, amount, description) before the final confir[illegible]<br>S16: Avoid multiple transactions when the system is slow. Solution: Disable the submissio[illegible] and display a message after the first submission: "The transaction is under progress processin[illegible] wait." |

| | | |
|---|---|---|
| | • P15: Lack of a preview system to confirm the payer and receiver: Before the final sending, the user cannot see a preview of the payer and receiver to ensure the correctness of the information.<br>• P16: It is possible to perform multiple transactions by mistake during system latency. When the banking system is slow, the user may hit the submit button several times, and duplicate transactions may occur. | |

| | | |
|---|---|---|
| Recognition rather than recall | • P1: Use of unfamiliar symbols: Unusual symbols are used for key application parts in which users must remember their meaning.<br>• P2: Lack of short instructions for button functions: Buttons and sections lack short and immediate explanations, and users must remember what each button does.<br>• P3: Need to remember complex steps: To perform complex transactions, the user needs to remember the steps because there are no visual guides or step-by-step explanations.<br>• P4: Do not display user information by default: Essential information, such as account numbers or balances for user selections, should be displayed directly and not hidden.<br>• P5: No drop-down list for frequently used choices: When entering information such as card numbers or frequently used accounts, the application does not suggest options, so the user has to remember what to enter.<br>• P6: Not displaying default information for repeated transactions: The user's previous information is not displayed, and they must remember and enter the information again.<br>• P7: Lack of additional explanations for banking terms: Using specialized banking terms without providing explanations and examples forces users to remember the meaning of these terms.<br>• P8: Users must re-enter information for similar transactions. The app will not make automatic suggestions for similar transactions. Users must enter all information manually for these transactions.<br>• P9: Lack of auto-completion: The application cannot enter information such as account numbers and saved names.<br>• P10: Clear images and icons are not used to identify sections: Different sections of the application are marked with text only, and clear images and icons are not used for quick identification.<br>• P11: Not displaying the access routes in the navigation: On different pages, the access routes or the user's current location in the application are not displayed, and the user has to remember which section he/she is in | S1:Simple, familiar icons: use well-known icons and clear text labels for essential buttons.<br>S2:Brief tooltips: short, straightforward tooltips or labels beneath buttons to quickly clarify th purpose.<br>S3:Step-by-Step Guides: Provide visual guides that break down complex processes into easy- follow steps.<br>S4:Default Display of Essential Information: Ensure critical information, such as account nun balance, is prominently displayed without extra clicks.<br>S5:Dropdown Menus for Frequent Selections: Include pre-filled options for commonly entere like card or account numbers, to streamline user input<br>S6:Default Values for Repeated Transactions: Automatically save and present information fro recent transactions for easy reuse.<br>S7:Simple Explanations of Banking Terms: Add brief definitions or examples for specialized banking terminology.<br>S8:Simple Explanations of Banking Terms: Provide brief, easy-to-understand definitions or e of specialized banking terminology to ensure users feel informed.<br>Autocomplete Functionality: Autocomplete is implemented for input fields, such as saved acc or card numbers.<br>S10:Clear and Relevant Icons: Create clear and relevant icons with supporting text to help use quickly identify sections and navigate the app.<br>S11:Relevant Icons: Create clear and relevant icons accompanied by supporting text to guide the correct sections. |

| | | |
|---|---|---|
| Flexibility and efficiency of use | P1: Lack of personal settings for quick access to frequently used sections: The application does not allow users to customize the home screen or create a shortcut to access frequently used sections.<br>P2: There are no shortcut options for frequent transactions. Users cannot create shortcuts or predefined templates for transactions they perform frequently.<br>P3: Lack of professional and beginner user modes: The application does not allow users to switch between a more straightforward mode for beginner users and a more advanced mode for professional users.<br>P4: Failure to provide different login methods: The application uses only one login method and does not provide options, such as fingerprint login or facial recognition.<br>P5: No language change option: Users cannot change the app language to other languages, which is challenging for bilingual or foreign users.<br>P6: Users cannot adjust the information display format, such as font size or color theme, according to their needs.<br>P7: No option to save recommended transactions: The application cannot save frequently used or frequent transactions as templates; users must enter the information each time.<br>P8: Limited quick access to various features: To perform various transactions or use banking tools, users must go through several steps and do not have quick access to all features.<br>P9: Lack of advanced search capabilities: This application does not offer advanced search to find specific transactions or information.<br>P10: Inability to manage multiple accounts simultaneously: The application cannot display and manage multiple bank accounts simultaneously; thus, users must log in to their accounts separately. | **S1: Possibility of personal settings with quick access**<br>**Each repetitive process can be easily arranged in a daily to-do list for older adults.**<br>**S2: Provide shortcut options for frequently used transactions.**<br>**The proposed method saves time by storing predefined templates for quick transactions [illegible]**<br>**S3: Providing beginner and professional modes**<br>**Adaptable modes for users of all experience levels. Seniors with more experience should [illegible]**<br>**increase the number of items used in the application, and beginners should place the mo[illegible]**<br>**straightforward number of operations on the page according to their ability.**<br>**S4: Supporting various login methods**<br>**Enhanced security and convenience with various login methods facilitate login techniqu[illegible]**<br>**fingerprinting, facial recognition, and dynamic passwords.**<br>**S5: Ability to change application language (ASL)**<br>**provide multilingual settings to support bilingual or foreign users.**<br>**S6: Information display settings based on user requirements**<br>**It adapts the application's font size, color scheme, and information presentation format.**<br>**S7: The ability to save suggested transactions**<br>**Capacity to save suggested and regular transactions as templates for later retrieval.**<br>**S8: Quick access to various features**<br>**Develop a toolbar or drop-down menu that provides single, direct access to primary tool[illegible]**<br>**functions.**<br>**S9: Advanced search for transaction information**<br>**To provide advanced search capabilities with filters, e.g., date, amount, and transaction [illegible]**<br>**S10: Simultaneous management of multiple bank accounts**<br>**Develop a means to view and control several bank accounts in a single user interface.** |

| | | |
|---|---|---|
| Esthetic and minimalist design | P1: Congested home page with many options: The home page is full of icons and different sections, making it crowded and difficult to navigate.<br>P2: Overuse of inconsistent colors and themes: Different parts of the app use different colors and themes, reducing visual harmony and simplicity.<br>P3: Displaying unnecessary details on the home page: The home page displays unnecessary information for the user, which can be confusing.<br>P4: Lack of white space for better readability: The design is too dense, with little space between elements, making it difficult to read and focus.<br>P5: Excessive use of decorative elements that do not add value: The application uses decorative icons or graphics that are not functional and clutter the user interface.<br>P6: Poor contrast between text and background: The color contrast between text and background is insufficient in some parts, making reading difficult.<br>P7: Inconsistent font sizes and types: Different app parts use different font sizes and styles, disrupting visual coherence.<br>P8: Too many steps to perform simple tasks: The design requires multiple steps to complete basic tasks, reducing the user experience's simplicityence.<br>P9: Lack of minimalistic icons and images: The icons and graphics used are overly complex or detailed, detracting from the app's minimalist look.<br>P10: Displaying too much information on one page: Some pages have excessive text or data, which overwhelms the user and makes the application challenging. | -S1: Designing a home page with a focus on priorities<br>Based on the needs of the elderly and the results of their interviews, the most frequently used should be placed in this dashboard section. For example, Mind games-frequent transactions<br><br>- S2: Harmonizing colors and themes<br>Text and background colors should be suitable for seniors to improve their vision. Instead of emphasizing multiple organizational colors, use blue to green for seniors.<br>- S3: Remove unnecessary information from the home page.<br>Information less than five modules with a memory density of 7+2 should be considered.<br>- S4: Create white space between elements.<br>Add appropriate white space to separate elements and increase readability.<br>- S5: Reduce the use of nonfunctional decorative elements.<br>Remove unnecessary graphic icons and use simple visual elements.<br>- S6: Improve text-background contrast<br>Choose high-contrast colors for the text and background, such as white. This ensures the desi[illegible] legibility and provides a comfortable experience for users.<br>- S7: Unify font sizes and types<br>Use a consistent font style and size to increase visual consistency up to 16 (more than the ave[illegible] size of 12).<br>- S8: Simplify processes and reduce steps<br>By optimizing the workflow, the number of clicks and steps required to complete simple task[illegible] reduced.<br>-S9: Simple, clear icons and images<br>Use minimal icons and images with minimal detail to increase focus and beauty.<br>- S10: Divide the information into multiple pages or sections.<br>Organize lengthy information into separate pages or tabs to avoid visual clutter. |

| | | |
|---|---|---|
| Help users recognize, diagnose, and recover from | P1: Failure to display error messages clearly: In the case of an error, the error message is not displayed clearly, and the user cannot understand the cause of the problem.<br>P2: Nonspecific and general error messages: Error messages are too general and lack sufficient detail, and the user cannot identify the exact reason for the error.<br>P3: Lack of guidance to correct the error: In the event of an error, the user is not given guidance to correct or fix the problem.<br>P4: Failure to record the date and time of the error: If the time and date of the error are not recorded, the user may be unable to track the problem.<br>P5: No recovery from payment errors: If a payment process error occurs, the user cannot simply redo the transaction and recover their information.<br>P6: No use of visual cues for error detection: When an error occurs, visual cues such as color changes or a specific icon are not used to identify the problem.<br>P7: No rollback options for incomplete tasks: If a transaction or operation is incomplete, there is no option to rollback or cancel it.<br>P8: Technical error messages for regular users: Technical error messages that are not understandable for regular users are presented instead of simple and understandable language.<br>P9: There is no notification system for the status of transactions. If a transaction is performed incorrectly or is delayed, the user is not notified to ensure its status. | S1: Display error messages, Explain the problem using simple and concise language, and sho<br>message in a clear and prominent location. You can also use an audio warning.<br>S2: Provide precise error messages that instill confidence and personalize the error messages<br>providing details about the cause of the problem, such as setting the text by itself.<br>S3: Add error troubleshooting tips. The error message should display clear, short steps to fix t<br>problem or complete the process as a pop-up or audio.<br>S4: Record the date and time of the error. Displaying the time and date of the error in the mes<br>critical for user follow-up and troubleshooting.<br>S5: Enable easy recovery from payment errors to provide a sense of security<br>Adding a “back” or “retry” button is essential to prevent users from re-entering information a<br>payment error, enhancing their experience and preventing frustration.<br>S6: Using visual cues to identify errors<br>Use graphic cues, such as color changes, warning icons, or highlighted lines, to indicate the lo<br>of the problem.<br>S7: Add an option to return or cancel incomplete operations.<br>The ability to cancel or return to the previous state if the transaction or operation remains inco<br>S8: Simplify the language of technical error messages and replace technical messages with si<br>and understandable terms for ordinary users.<br>S9: Create a transaction status notification system and design one to send notifications or aler<br>the successful or unsuccessful status of transactions. |

| | | |
|---|---|---|
| Help and documentation | p1: Lack of Easy Access to the Help Section: The help section is difficult to find, which makes it difficult for users to seek help when encountering issues.<br>p2: Inadequate Documentation: The documentation, when available, does not offer sufficient detail or clarity on how to use the application effectively.<br>p3: Lack of Searchable Content in the Help Section: Users struggle to locate specific help topics or solutions in the help section without a search function.<br>p4: Absence of Step-by-Step Guides: Users lack clear instructions for simple and complex tasks, making the app more challenging.<br>P5: Absence of Video Tutorials: The app misses visual aids, such as video tutorials, that could enhance understanding and user experience.<br>p6: Outdated or Irrelevant Help Content: The help documentation is not current, which can lead to confusion, significantly if features or processes have changed.<br>p7: Lack of Easy Response to Frequently Asked Questions (FAQ): The absence of an FAQ section means that users cannot easily find answers to common queries, leading to frustration.<br>p8: Absence of In-App Textual Help: The app does not provide on-the-spot guidance or tooltips in different sections, leaving users to figure things out independently.<br>p9: Lack of Support for Different Languages: The help section is only available in one language, which could limit the accessibility of users from different linguistic backgrounds. - | S1: Make the Help section easy to access.<br>Emphasizing the significance of adding a 'Help' or 'Support' button to the main menu or displa prominently on the home screen. This ensures quick access to assistance, thereby enhancing u experience.<br>- S2: Provide complete and accurate documentation<br>Prepare comprehensive documentation of all features and processes in sufficient detail. Please this option in the menu so users can refer to it when necessary.<br>-S3: Add search functionality to Help.<br>An internal search engine can be added to the Help section to find specific topics or solutions quickly.<br>- S4: Design step-by-step guides<br>Provide step-by-step visual and text instructions for performing simple and complex tasks.<br>- S5: Add training videos.<br>Create short and simple videos to teach the features and processes of the application.<br>- S6: Regular content updates<br>Continuously reviewing and updating the documentation and Help section to coordinate with application changes.<br>- S7: Add a Frequently Asked Questions (FAQ) section<br>Design an FAQ section to quickly answer common user questions and provide guidance on h use it.<br>- S8: Provide in-app text assistance<br>Add help tips or small widgets (Tooltips) to different program parts for real-time guidance.<br>- S9: Multilingual support in the help section.<br>Provide help content in multiple languages for users with different languages, such as Persian Turkish or Kurdish, for use by different cultural groups. |

| | | |
|---|---|---|
| Customization options | p1: Limited access to the help section: There is no immediate access to the help section and no timely access to guidance when needed.<br>p2: Inadequate documentation: Existing documentation does not provide enough information or guidance to state and use the application effectively.<br>p3: Inability to search in the help section: Users cannot search in the help section for specific keywords or subjects, and therefore, both search and retrieval become tedious and cumbersome.<br>p4: Inadequacy of guides: There are no specific, explicit guides for following through with specific actions, so users have to interpret the processes individually.<br>p5: Inadequacy of instruction videos: There are no instruction videos or graphical aids to enable users to understand and use the application effectively.<br>p6: Obsolete and irrelevant help documents: The help documents have not been updated, so the information in them is outdated and irrelevant to current application releases.<br>p7: Inability to readily access frequently asked questions (FAQs): There is no FAQ section, and therefore, there is no access to immediate information for frequently asked questions.<br>p8: Inability to access in-app textual guidance: There is no in-app guidance in terms of help and immediate textual guidance in sections, and therefore, no ease of access to guidance for users when accessing and using the application. | S1: Personalizable menu items: Users can personalize menu items in the Help menu by marki<br>frequently visited items for quick access.<br>S2: Personalized documentation: Providing relevant documentation according to a user's level<br>expertise (novice or expert) maximizes the effectiveness of the information delivered.<br>S3: Personalized search performance:<br>Enhance user productivity through sophisticated search filtering capabilities (e.g., by category<br>keyword, urgency) in the Help section, allowing users to access information easily and in a ti<br>manner.<br>S4: Interactive Step-by-step Guides<br>Empower users to actively direct their educational processes by creating and storing personali<br>guides for complex processes, allowing them to have control over their educational path.<br>S5: On-Demand Video Tutorials<br>Facilitate educational progression for users through a collection of on-demand tutorial videos,<br>allowing them to save or download the most relevant videos for future use.<br>S6: Customizable FAQ Builder<br>Create a dynamic FAQ section that allows users to rank current questions according to releva<br>S7: Contextual Help Tooltips<br>Allow users to enable immediate help tooltips in the application and choose a theme for guida<br>presentation.<br>S8: Multilingual and Display Options<br>The feature introduces multilingualism in addition to options for customizing fonts or display<br>in the Help section, enhancing users' comfort and overall usability. |

| | | |
|---|---|---|
| Physical ergonomics and interaction | **p1: Inappropriate Buttons and Options**: Buttons or options are too small or close together, causing incorrect selection.<br>**p2: Inability to use ergonomic settings**: Users cannot change the application's settings, such as the font size or the spacing of elements, to their liking.<br>**p3: Incompatibility with One-Handed Use**: The application's design does not make it easy for users to use it with one hand.<br>**p4: Cluttered and Complicated Pages**: The design of the app's pages is cluttered and complicated, which confuses users.<br>**p5: Absence of Tactile or Audio Feedback**: When performing transactions, the user is not given appropriate tactile or audio feedback.<br>**p6: Improper Lighting and Screen Reflection**: The screen is not easily visible in environments with high or low light.<br>**p7: Not Paying Attention to the Needs of Elderly or Visually Impaired Users**: The application does not have ergonomic settings for elderly or visually impaired users.<br>**p8: Difficulty Entering Financial Information**: It is challenging to enter sensitive information, such as card numbers or passwords, in the application | S1: Redesigned Buttons and Spacing<br>We enlarged buttons and increased the spacing between them to reduce accidental selecti [illegible] improve usability.<br>S2: Customizable Display Settings<br>Provide users with options to adjust font size, line spacing, button size, and screen brightnes [illegible] their preferences.<br>S3: One-handed Mode<br>Implement a dedicated one-handed mode by repositioning interactive elements to mal [illegible] accessible with the thumb for easier use.<br>S4: Simplified and Minimalist Layout<br>Redesign cluttered pages with clear groupings and fewer elements to create a more user-frier [illegible] organized interface.<br>S5: Provide haptic and audio feedback<br>Add haptic vibrations or audio cues for essential actions like confirming transactions or [illegible] buttons to increase engagement.<br>S6: Adaptive display settings for brightness control<br>High contrast mode and automatic brightness adjustment to improve visibility under different [illegible] conditions.<br>S7: Accessibility features of visually impaired elderly and visually impaired users<br>Integrate voice guidance, more extensive text options, and screen reader compatibility for el [illegible] visually impaired users.<br>S8: Simplified financial information entry<br>To facilitate data entry, introduce features like OCR (optical character recognition) for scann [illegible] numbers, auto-complete for frequent entries, and voice input for passwords. |
| **T** | **135** | **135** |
